# Effects of high glucose on human umbilical vein endothelial cell permeability and myosin light chain phosphorylation

**DOI:** 10.1186/s13098-015-0098-0

**Published:** 2015-11-14

**Authors:** Xiao-Yan Zhao, Xiao-Fang Wang, Ling Li, Li Zhang, De-Liang Shen, Dan-Hua Li, Qiang-Song Jin, Jin-Ying Zhang

**Affiliations:** Department of Cardiology, First Affiliated Hospital of Zhengzhou University, No.1 Jianshe East Road, 450052 Zhengzhou, China

**Keywords:** Human umbilical vein endothelial cell, Permeability, Myosin light chain, High glucose, RhoA/ROCK pathway

## Abstract

**Background:**

Diabetes mellitus is one of the most important risk factors for atherosclerosis. However, the mechanisms underlying high-glucose-induced atherosclerosis remain unclear. This study was designed to observe the effects of high-glucose stimulation on the permeability of cultured human umbilical vein endothelial cells (HUVECs), and to explore the effects of RhoA–Rho-associated protein kinase (ROCK) signal transduction pathway activation and myosin light chain (MLC) phosphorylation.

**Methods:**

HUVECs were cultured in conventional M199 medium to produce endothelial cell monolayers, and stimulated with high-glucose-M199 medium. The transmembrane transport of dextran and THP-1 cells and levels of MLC phosphorylation were measured. The effects of blocking the RhoA-ROCK pathway using dnRhoA or the ROCK inhibitor Y27632 on dextran and THP-1 transport and MLC phosphorylation were observed.

**Results:**

Transendothelial migration of dextran and THP-1 cells were significantly increased by stimulation of HUVEC monolayers with high glucose (*P* < 0.05). This effect was attenuated by treatment with dnRhoA or Y27632.

**Conclusion:**

High-glucose stimulation upregulated MLC phosphorylation and increased endothelial permeability by activating the RhoA-ROCK signaling pathway in HUVECs in vitro.

## Background

**Fig. 1:**
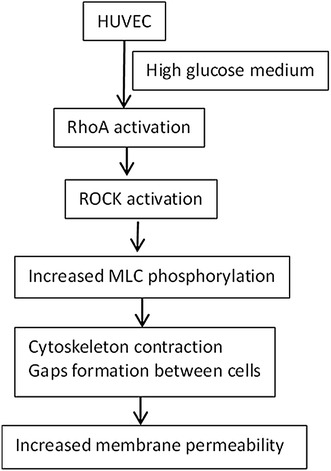
Presumed high glucose-induced cellular pathway linking to the Rho-dependent steps

Diabetes mellitus is one of the most important risk factors for atherosclerosis. However, the mechanisms underlying high-glucose-induced atherosclerosis remain unclear. Previous studies have demonstrated that high glucose can destroy endothelial function [1–3], which is an initiating condition for atherosclerosis. Rho family GTPases, especially RhoA, play an important role in maintaining and adjusting endothelial barrier function [4, 5]. Activation of RhoA enhances the activity of its downstream Rho kinase [Rho-associated protein kinase (ROCK)], which in turn induces myosin light chain (MLC) phosphorylation [6]. MLC phosphorylation results in the formation of gaps between adjacent cells through cytoskeleton contraction, resulting in increased membrane permeability. Figure 1 shows the presumed high-glucose-induced cellular pathway linking to the Rho-dependent steps.

In this study, we cultured human umbilical vein endothelial cells (HUVECs) in vitro, and observed the effects of high-glucose stimulation on endothelial monolayer permeability. We also examined the effects of blocking the RhoA-ROCK signaling pathway using dominant negative RhoA (dnRhoA) and the specific ROCK inhibitor Y27632 on the high-glucose-induced increase in endothelial permeability.

## Methods

### Cells and reagents

HUVECs (ATCC, USA) were incubated in M199 (Gibco, USA) medium supplemented with low-serum growth supplement (Gibco, USA), 10-mg/l gentamicin, and 0.25-mg/l amphotericin in a 5 % CO_2_ incubator at 37 °C and 95 % humidity. Sixth-passage HUVECs were seeded in 100-mm diameter Petri dishes prior to further treatments. When the cells reached confluence, they were incubated with M199 without low-serum growth supplement for 10 h. Cells in the stationary phase were used for experiments. THP-1 cells (ATCC, USA) were incubated with RPMI-1640 medium containing 50 μmol/l 2-mercaptoethanol, 10 % fetal bovine serum, 100 IU/ml penicillin, and 100 μg/ml streptomycin in a 5 % CO_2_ incubator at 37 °C and 95 % humidity. The glucose concentration of the ordinary M199 medium was 5.6 mmol/l. High-glucose medium was prepared by adding d-glucose to M199 to reach a final concentration of 25 mmol/l. Cell lysates were prepared using 25 mmol/l HEPES, pH 7.5, 150 mmol/l NaCl, 1 % NP-40, 10 mmol/l MgCl_2_, 1 mmol/l ethylenediamine tetraacetic acid, 2 % glycerol, 10 μg/ml leupeptin, 10 μg/ml aprotinin, 1 mM NaF, and 1 mM sodium vanadate. Dominant negative RhoA cDNAs (DN: N19) were purchased from UMR Resource Center (USA) and Y27632 were purchased from Selleck Chemicals (USA).

### Experimental groups

Cells were divided into six groups: control group, high-glucose group, dnRhoA group, Y27632 group, high-glucose + dnRhoA group, and high-glucose + Y27632 group. Control HUVECs were incubated with ordinary M199 medium (glucose concentration 5.6 mmol/l). HUVECs in the high-glucose groups were incubated with high-glucose medium. HUVECs in the dnRhoA groups were incubated with medium containing dnRhoA 10 μl/10 ml and HUVECs in the Y27632 groups were incubated with medium containing 10 µmol/L Y27632 in DMSO.

### Measurement indexes

#### Transendothelial dextran transfer

HUVECs were grown in a Transwell system using 0.4-μm micropore polycarbonate membranes (Millipore, USA) until complete confluence, resulting in the formation of an endothelial cell monolayer barrier. Fluorescein isothiocyanate-labeled dextran (MW 70,000; Seebio, Shanghai, China) 100 mg/l was added to the bottom well, and the relevant medium was added to the bottom and top wells, according to the different groups. After 2 h of incubation, 100 μl of medium was removed from the bottom and top wells and the fluorescence was determined using a fluorescence spectrometer. The rate of transendothelial dextran transfer was calculated as %dextran/h/cm^2^.

#### Transmembrane migration of THP-1 cells

The back of Transwell with 8.0-μm micropores (Millipore, USA) were coated with 50 % Matrigel. HUVECs were seeded on the other side of the Transwell membrane until complete confluence and formation of an endothelial monolayer barrier. THP-1 cells in the stationary phase were labeled with 2′,7′-bis-(2-carboxyethyl)-5-(and-6)-carboxyfluorescein acetoxymethyl ester (Fanbo, Beijing, China) (1 × 10^5^ cells/well) and seeded on the surface of the HUVECs, which were incubated with medium according to the different groups for 6 h. After treatment, nonmigrated cells were removed from the upper side of the membrane with cotton swabs, and the cells on the lower surface of the membrane were fixed, stained and observed under an inverted microscope.

#### MLC phosphorylation

HUVECs were divided into the above six groups and incubated for 6 h. Proteins were extracted from cell lysates and protein concentrations were determined using bovine serum albumin assay. MLC protein and phosphorylation levels were measured by western blotting using phosphorylated MLC antibody (1:500; Cell Signaling) as the primary antibody (Thr18/Ser19) and horseradish peroxidase-labeled goat anti-rabbit antibody (1:3000; Santa Cruz) as the secondary antibody. After visualization, proteins were eluted from the polyvinylidene fluoride membrane, followed by MLC antibody hybridization and visualization. Total protein levels of MLC were determined. The intensities of the gray bands were read using Image J software. The ratio of p-MLC to MLC in each group was calculated. Glyceraldehyde 3-phosphate dehydrogenase (GAPDH) was used as an internal reference.

### Statistical analysis

All data were analyzed using SPSS 13.0 software. Mean values were compared among multiple groups using one-way analysis of variance and least significant difference *t*-tests. A value of *P* < 0.05 was considered statistically significant.

## Results

### Transendothelial transfer of dextran and THP-1 cells

Transendothelial transport of dextran and THP-1 cells were significantly increased (*P* < 0.01) by high-glucose stimulation. The administration of dnRhoA or the ROCK inhibitor Y27632 significantly attenuated the effects of high-glucose medium (Table 1).

**Table 1:** Quantitative analyses of transendothelial transfer of dextran and THP-1 cells and MLC phosphorylation

Group	n	Transendothelial transfer rate of dextran (%dextran/h/cm^2^)	THP-1 transendothelial migration (cell/HP)	MLC phosphorylation
Control	5	9.09 ± 0.49	7.00 ± 1.22	0.00 ± 0.00
High glucose	5	16.72 ± 1.51^*^	30.00 ± 6.44^*^	0.73 ± 0.28^*^
dnRhoA	5	10.31 ± 1.97	10.00 ± 1.87	0.16 ± 0.02
Y27632	5	9.98 ± 0.78	9.00 ± 1.58	0.23 ± 0.03
High-glucose + dnRhoA	5	13.04 ± 0.91^#^	17.00 ± 1.87^*,#^	0.34 ± 0.04^#^
High glucose + Y27632	5	10.96 ± 0.39^#^	18.20 ± 1.79^*,#^	0.46 ± 0.03^#^
*F*		29.503	39.210	24.205
*P*		0.000	0.000	0.000

* *P* < 0.01 vs. control group; ^#^ *P* < 0.01 vs. high-glucose group

### MLC phosphorylation

**Fig. 2:**
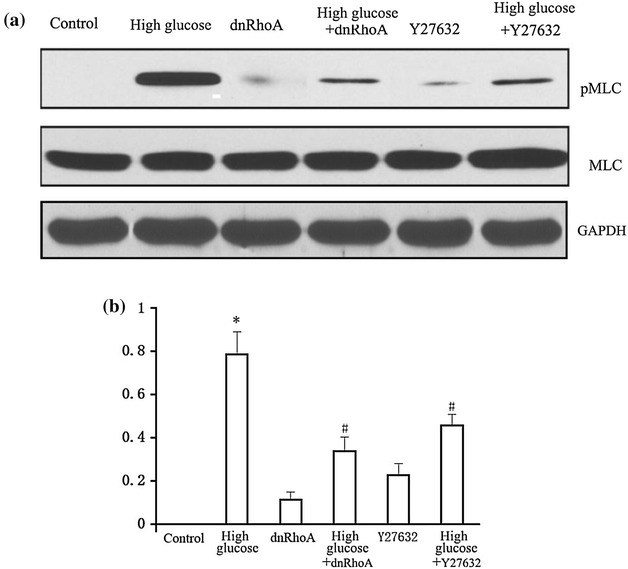
Effects of dnRhoA and Y27632 on high-glucose-induced MLC phosphorylation. MLC phosphorylation was not detectable in HUVECs cultured at normal glucose concentration but was significantly increased after stimulation with high glucose. MLC phosphorylation was significantly reduced by blocking RhoA and ROCK activities with dnRhoA and Y27632, respectively. Glyceraldehyde 3-phosphate dehydrogenase (GAPDH) was used as an internal reference. **a** Western blot assay; **b** bar graph. *P < 0.01 vs. control group; ^#^ *P* < 0.01 vs. high-glucose group

There was no significant difference in MLC protein expression among the groups and MLC phosphorylation was not detectable in HUVECs cultured at normal glucose concentration. However, MLC phosphorylation was significantly increased by stimulation with high glucose. Administration of dnRhoA or the ROCK inhibitor Y27632 significantly suppressed the high-glucose-induced MLC phosphorylation (Fig. 2; Table 1).

## Discussion

Atherosclerosis is a complex chronic inflammatory process associated with excessive inflammation and lipid accumulation [7]. Its major initiating factor is local endothelial cell injury. Endothelial cell injury can result in increased endothelial permeability and transendothelial transport of lipids and cellular blood components to the intima [8]. Initial fatty streaks form after a series of processes such as inflammatory reaction, oxidative stress, and smooth muscle cell migration and proliferation. These fatty streaks subsequently develop into plaques, including unstable plaques, which may then rupture and cause clinical events. The process is thus strongly associated with inflammation and endothelial dysfunction. Diabetes mellitus is a major risk factor for atherosclerosis. Although the mechanisms underlying high-glucose-induced atherosclerosis remain unclear, previous studies have confirmed that high glucose can destroy endothelial function, which is the initiating factor for atherosclerosis.

In this study, we cultured HUVECs in vitro to create single membranous structures. Transmembrane transport of dextran and THP-1 cells were increased by high-glucose stimulation, confirming the destructive effects of high glucose on endothelial function at the cellular level. These findings may explain why diabetic patients are at high risk of developing atherosclerosis.

The integrity of vascular endothelial function depends mainly on the normal functioning of cytoskeletal proteins and intercellular connections. A variety of physical factors (such as hemodynamic changes) and chemical factors (such as inflammatory cytokines) can affect expression levels of cytoskeletal proteins and intercellular junction proteins through specific signal transduction pathways, leading to endothelial dysfunction and increased permeability. Protein phosphorylation represents an important mechanism for regulating cytoskeletal protein function [9–11]. MLC phosphorylation can induce cytoskeleton contraction, thus opening gaps between cells and causing increased permeability [12–14]. In the present study, although MLC protein expression levels in HUVECs were unaffected by high-glucose stimulation, MLC phosphorylation levels were increased, indicating a role for MLC phosphorylation in high-glucose-induced endothelial permeability.

Previous studies confirmed that the Rho family GTPases, especially RhoA, played a crucial role in maintaining and regulating endothelial barrier function [4, 5]. RhoA activation enhanced activity of downstream ROCK, the key function of which is to induce MLC phosphorylation [6]. MLC phosphorylation in turn could increase endothelial permeability via cytoskeleton contraction and subsequent loss of endothelial cell adhesion and connections [3]. The results of the current study demonstrated that MLC phosphorylation levels and endothelial permeability were indeed increased by culture of HUVECs in high-glucose medium. However, this high-glucose-induced permeability was partially inhibited and MLC phosphorylation levels noticeably diminished by suppression of the RhoA-ROCK signaling pathway with dnRhoA or Y27632. These findings suggest that high-glucose stimulation upregulated MLC phosphorylation and destroyed endothelial function by activating the RhoA-ROCK system. The mechanism responsible for the high-glucose-induced increase in endothelial permeability may thus be as follows: high glucose → RhoA activation → ROCK activation → MLC phosphorylation → cytoskeleton contraction → gap between cells → permeability increase.

In addition to ROCK, MLC kinase is also an upstream regulatory kinase of MLC phosphorylation. We therefore used a control group to account for the possible effects of Y27632 and dnRhoA solvent on MLC kinase activation, which could affect MLC phosphorylation levels. The results showed that Y27632 and dnRhoA alone could induce a small amount of MLC phosphorylation, but the mechanisms responsible for this phenomenon are unclear. Further investigations are needed to determine if Y27632, dnRhoA, or their solvent components affect MLC kinase activity or intracellular calcium concentrations.
